# QKD Based on Symmetric Entangled Bernstein-Vazirani

**DOI:** 10.3390/e23070870

**Published:** 2021-07-07

**Authors:** Michael Ampatzis, Theodore Andronikos

**Affiliations:** Department of Informatics, Ionian University, 7 Tsirigoti Square, 49100 Corfu, Greece; p16abat@ionio.gr

**Keywords:** quantum cryptography, quantum key distribution, the Bernstein-Vazirani algorithm, EPR pairs, quantum entanglement, quantum information theory

## Abstract

This paper introduces a novel entanglement-based QKD protocol, that makes use of a modified symmetric version of the Bernstein-Vazirani algorithm, in order to achieve secure and efficient key distribution. Two variants of the protocol, one fully symmetric and one semi-symmetric, are presented. In both cases, the spatially separated Alice and Bob share multiple EPR pairs, each one qubit of the pair. The fully symmetric version allows both parties to input their tentative secret key from their respective location and acquire in the end a totally new and original key, an idea which was inspired by the Diffie-Hellman key exchange protocol. In the semi-symmetric version, Alice sends her chosen secret key to Bob (or vice versa). The performance of both protocols against an eavesdroppers attack is analyzed. Finally, in order to illustrate the operation of the protocols in practice, two small scale but detailed examples are given.

## 1. Introduction

In the course of the last century, the scientific community experimented with different ideas and forms of computation, trying to harness the power of nature and create machines that allowed us to process immeasurable amounts of information in mere seconds, thus radically changing the world around us in the span of a few decades. However, in the present era classical computers are reaching a point where it will be infeasible to substantially enhance their efficiency due to the physical limitations of transistors. This has started a new incentive to resurrect previous attempts concerning research of new types of computation. Out of all the different proposals for a viable substitute to classical computing, undoubtedly the most promising of them all is quantum computation, mainly due to the fact that it allows the exploitation of the most fundamental properties of physics.

### 1.1. Related Work

As technology comes closer to the realization of this goal, it appears that certain profound adaptations regarding different branches of computer science need to take place in order to achieve a smoother transition from the classical to the quantum era. One of the most important such branches is the field of cryptography, due to the vulnerability of the current security algorithms against quantum computers [1,2]. This inherent weakness in the modern security protocols and the race for building a resilient security infrastructure against quantum computers [3] before they become a reality, were the two catalysts that resulted in a schism of the field into two sub-fields, which are based on two different philosophies and ideologies. The first sub-field, known as post-quantum cryptography or quantum-resistant cryptography, relies on the complexity of mathematics as its security basis. It is an attempt to develop cryptographic systems that are secure against both quantum and classical computers and can also be interpreted within the already existing communications protocols and networks. The second sub-field, which is called quantum cryptography, is being built upon the implementation of the properties of quantum mechanics and, thus, takes advantage of nature’s own fundamental laws in order to achieve security.

The sub-field of quantum cryptography, on which the primary interest of the current paper lies upon, has seen enormous growth of both theoretical and practical nature. Two landmark papers, the BB84 protocol [4] and the E91 protocol [5], were the first papers that proved that key distribution between two parties relying on the properties of quantum mechanics was possible. These two protocols have established the two schemes that all quantum key distribution (QKD) protocols are based on, the *prepare-and-measure-based scheme* and the *entanglement-based scheme*. After the publications of these two protocols, a plethora of interesting proposals for different QKD protocols based on these two schemes were suggested, further expanding the field on a theoretical level. At the same time, some truly remarkable real life implementations of some protocols were demonstrated as in [6,7,8,9,10,11]. These implementations have demonstrated that quantum cryptography is not just a mere theoretical experiment, but a possible reality in the near future.

Over the last few years, there was a noticeable increase in the effort to find new viable applications for well-known quantum algorithms, such as the Deutsch-Jozsa algorithm [12], the Bernstein-Vazirani algorithm [13] and Simon’s periodicity algorithm [14]. Many of these proposals have been made in the field of quantum cryptography, using these algorithms as viable QKD protocols [15,16,17]. Motivated from these attempts, this paper proposes two novel variants of an entanglement-based QKD protocol that makes use of the Bernstein-Vazirani algorithm. The novelty of this work lies on the fact that it uniquely combines some key ingredients. Starting with entanglement, which is an integral part of the protocol, the corresponding qubits in Alice and Bob’s input registers are maximally entangled. Thus, the proposed protocols exhibit all the inherent advantages that an entanglement-based QKD protocol provides in terms of security against an eavesdropper, as first demonstrated in the E91 protocol [5]. Additionally, the Bernstein–Vazirani algorithm [13], a fast and useful quantum algorithm that guarantees the creation of the key using just one application of the appropriate function, is used in a critical manner. Furthermore, the fully symmetric variant is inspired by the Diffie-Hellman idea [18] of deriving the final key from a random combination of two separate keys. This idea is not just cosmetic, as the ability to obtain a key that neither Alice or Bob know from the start, adds an additional layer of security, further improving the strength of the protocol. Finally, the proposed protocol can be implemented in two versions: the fully symmetric version and the semi-symmetric one. In the fully symmetric variant, both Alice and Bob can input their tentative secret keys from their respective locations and acquire in the end a totally new and original key. In the semi-symmetric one, Alice (alternatively Bob) constructs the secret key that she (or he) communicates securely to the other party.

The protocol is described as a quantum game, which despite the rather playful name, it is another noteworthy field that has emerged due to the transition to the quantum era and is used to address difficult and interesting problems within the quantum realm. This approach was chosen in an effort to make the presentation more mnemonic and easier to follow, due to the close connection that both fields share and the fact that any cryptographic situation can be conceived as a game between the two fictional heroes Alice and Bob, who play the roles of two remote parties that are trying to communicate, and the enemy Eve who tries to eavesdrop the conversation, a case which becomes apparent with the quantum game of coin tossing and the BB84 protocol [4,19] and references therein. This situation has been generalized in [20] to quantum dice rolling. For the reader striving for a more rounded understanding of the connection of the two fields, one can start with the two important works in the field of quantum game theory dating back to 1999, which were instrumental for the creation of the field: Meyer’s PQ penny flip game [21], which can be regarded as the quantum analogue of the classical penny flip game, and the introduction of the Eisert-Wilkens-Lewenstein scheme [22] that is widely used in the field. Regarding the PQ penny flip game, some recent results can be found in [23,24], were its connection to the dihedral groups was established. As for the Eisert-Wilkens-Lewenstein scheme, it proved fruitful in providing many interesting results. For example, it led to quantum adaptations of the famous prisoners’ dilemma in which the quantum strategies are better than any classical strategy [22], as well as extensions of the classical repeated prisoners’ dilemma conditional strategies to a quantum setting [25].

### 1.2. Organization

The paper is structured as follows. Section 1 provides a brief introduction to the subject and gives the most relevant references. Section 2 introduces and explains the tools used for the formulation of the protocols in this article. Section 3 presents and thoroughly analyzes the fSEBV and sSEBV protocols, so that their functionality can be completely understood. Section 4 contains two detailed examples, one for each protocol, to demonstrate their operation. Finally, Section 5 summarizes the proposed protocols and discusses their potential applications in various situations.

## 2. Preliminaries

### 2.1. Quantum Entanglement and Bell States

Quantum entanglement is one of the fundamental principles of quantum mechanics and can be described mathematically as the linear combination of two or more product states. The Bell states are specific quantum states of two qubits, sometimes called an EPR pair, that represent the simplest examples of quantum entanglement. From the perspective of quantum computation, an EPR pair can be produced by a circuit with two qubits, in which a Hadamard gate is applied to the first qubit and subsequently a CNOT gate is applied to both qubits. These states can be elegantly described by the following equation taken from [26].

(1) $$\begin{array}{c} |\beta_{x,y}\rangle =\frac{|0\rangle |y\rangle +{\left(-1\right)}^{x}|1\rangle |\bar{y}\rangle }{\sqrt{2}}\;, \end{array}$$

where $|\bar{y}\rangle$ is the negation of |y〉.

In a more detailed manner, the Bell states can be described as follows.

(2) $$\begin{array}{c} |\Phi^{+}\rangle =|\beta_{00}\rangle =\frac{|0\rangle |0\rangle +|1\rangle |1\rangle }{\sqrt{2}} \end{array}$$

(3) $$\begin{array}{c} |\Phi^{-}\rangle =|\beta_{10}\rangle =\frac{|0\rangle |0\rangle -|1\rangle |1\rangle }{\sqrt{2}} \end{array}$$

(4) $$\begin{array}{c} |\Psi^{+}\rangle =|\beta_{01}\rangle =\frac{|0\rangle |1\rangle +|1\rangle |0\rangle }{\sqrt{2}} \end{array}$$

(5) $$\begin{array}{c} |\Psi^{-}\rangle =|\beta_{11}\rangle =\frac{|0\rangle |1\rangle -|1\rangle |0\rangle }{\sqrt{2}} \end{array}$$

The main advantage of quantum entanglement is that if one qubit of the pair is measured, then the other will collapse immediately despite the distance between the two. This unique characteristic of quantum entanglement can be used on quantum key distribution as first described by Ekert in the E91 protocol. Therefore, in order to achieve quantum key distribution, multiple EPR pairs will be needed. For this reason, the mathematical representation of multiple EPR pairs will be expedient. If one starts with the entangled Bell state $|\Phi^{+}\rangle$, which can be cast as

(6) $$\begin{array}{c} |\Phi^{+}\rangle =\frac{1}{\sqrt{2}}\left({|0\rangle }_{A}{|0\rangle }_{B}+{|1\rangle }_{A}{|1\rangle }_{B}\right)\;, \end{array}$$

some easy computations show that

(7) $$\begin{array}{cc} {|\Phi^{+}\rangle }^{⊗n}\; & =\frac{1}{\sqrt{2^{n}}}\sum_{x\in {\left\{0,1\right\}}^{n}}{|x\rangle }_{A}{|x\rangle }_{B}\;, \end{array}$$

which will be required in the presentation of Section 3.

### 2.2. A Brief Description of the Bernstein-Vazirani Algorithm

Regarded as one of the earliest quantum algorithms, along with the Deutsch-Josza algorithm and Simon’s periodicity algorithm, the Bernstein-Vazirani algorithm, first introduced by Ethan Bernstein and Umesh Vazirani, can be considered to be a useful extension of the Deutsch-Josza algorithm, due to the fact that it was directly inspired by it and shared multiple common characteristics on both structure and implementation. Yet, despite the similarities, it has proved its value by demonstrating that the superiority of a quantum computer can be successfully used for more complex problems than the Deutsch-Josza problem.

The Bernstein-Vazirani problem can be described as the ensuing game between two players, namely Alice and Bob, who are spatially separated. Alice in Athens is corresponding with Bob in Corfu using letters. Alice starts the game by selecting a number *x* from 0 to $2^{n}-1$ and mails its binary *n*-bit representation x to Bob. After Bob receives this message, he calculates the value of some function

(8) $$\begin{array}{c} f:\left\{0,1,\cdots ,2^{n}-1\right\}\to \left\{0,1\right\}\;, \end{array}$$

and replies with the result, which is either 0 or 1. The rules of the game dictate that Bob must use a function $f_{s}\left(x\right)$, where $s=s_{n-1}\cdots s_{1}s_{0}$ and $x=x_{n-1}\cdots x_{1}x_{0}$ are *n*-bit binary numbers representing integers in the range $0,1,\cdots ,2^{n}-1$, such that

(9) $$\begin{array}{c} f_{s}\left(x\right)=s\cdot x\;\mathrm{mod}\;2\;. \end{array}$$

The inner product modulo 2 is defined as

(10) $$\begin{array}{c} s\cdot x\;\mathrm{mod}\;2=s_{n-1}x_{n-1}⊕\cdots ⊕s_{0}x_{0}\;, \end{array}$$

where ⊕ is the exclusive-or operator. Therefore, the function is guaranteed to return the bitwise product of Alice’s input x with a secret key s that Bob has chosen. Alice’s goal in this game is to determine with certainty the secret key s that Bob has picked, corresponding with him as little as possible. How fast can she succeed?

In the *classical* version of this problem, Alice can find the secret key s by taking advantage of the nature of the function $f_{s}\left(x\right)$ and, in particular, by sending Bob the inputs shown in Table 1.

In that way, Alice will discover a bit of the string s (the bit $s_{i}$) with each query she sends. For example, with x=10⋯0 she can obtain the most significant bit of s, with x=01⋯0 she will find the next most significant bit of s, and by following the same procedure, when she reaches x=00⋯1, she will have finally managed to reveal the entire string s. Despite, the efficiency of this method, Alice is still limited by sending to Bob only one query at a time. Therefore, the best possible classical scenario requires from her to correspond with Bob at least *n* times, in order for her to succeed in her goal.

By observing the core attributes of the aforementioned game, we can divide it into the following three big steps, which are:

- Alice provides an input,
- Bob applies the function $f_{s}\left(x\right)$, and
- after multiple repetitions of the previous two steps, Alice is finally able to reveal the secret key s.

It can be seen from the above steps that the game can easily become more efficient by implementing certain tools from quantum mechanics. If Alice and Bob were able to exchange information with the use of qubits instead of classical bits, then Alice could send the superposition of these qubits to Bob with only one message. Furthermore, if Bob was using a unitary transformation $U_{f}$ instead of a function $f_{s}\left(x\right)$, then Alice would be able to achieve her goal with only one communication.

**Figure 1:**
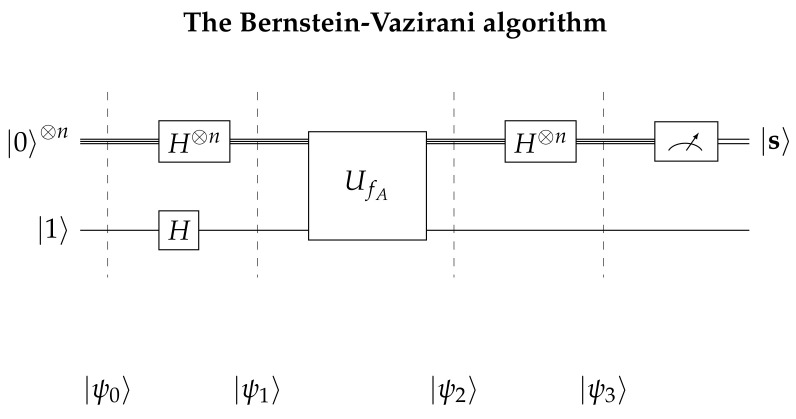
This figures gives a schematic representation of the Bernstein-Vazirani algorithm.

The *quantum* version of the Bernstein-Vazirani algorithm, can be described by the following quantum game. The game initially starts with Alice preparing two quantum registers, one of size *n* to store her query in and one of size 1, in which Bob will store his answer in. We will refer to these registers as Alice’s input and output registers, respectively. Next, she applies the Hadamard gate to every qubit, in order to acquire the even superposition state of each register and then she sends both registers to Bob. Right after Bob receives the contents of the registers, he applies the unitary transform $U_{f}$ and sends them back to Alice. In the final stage of the game, Alice concludes the algorithm by measuring her input register and obtaining the secret key s. The whole process of the game, is summarized in Figure 1 below.

Now, in order to obtain a better understanding of the nature of the algorithm, let us examine the evolution of the quantum states more closely. First, Alice starts with the initial state

(11) $$\begin{array}{c} |\psi_{0}\rangle ={|0\rangle }^{⊗n}|1\rangle . \end{array}$$

The *n* qubits of her input register are all prepared at state |0〉 and the qubit of the output register is prepared at state |1〉. Next, Alice applies the Hadamard transform to both registers and the state becomes

(12) $$\begin{array}{c} |\psi_{1}\rangle =\frac{1}{\sqrt{2^{n}}}\sum_{x\in {\left\{0,1\right\}}^{n}}|x\rangle \left(\frac{|0\rangle -|1\rangle }{\sqrt{2}}\right). \end{array}$$

The derivation of the previous equation is based on the fact that

(13) $$\begin{array}{c} H^{⊗n}{|0\rangle }^{⊗n}=\frac{1}{\sqrt{2^{n}}}\sum_{x\in {\left\{0,1\right\}}^{n}}^{}|x\rangle , \end{array}$$

a standard result in the literature (for its derivation see [26,27]). At this point the input register is in an even superposition of all possible states and the output register is in an evenly weighted superposition of |0〉 and |1〉. Thus, Alice is now ready to send both registers to Bob so he may apply the function $f_{s}\left(x\right)$ using

(14) $$\begin{array}{c} U_{f}:|x,y\rangle \to |x,y⊕f(x)\rangle \;, \end{array}$$

which results in the next state

(15) $$\begin{array}{c} |\psi_{2}\rangle =\frac{1}{\sqrt{2^{n}}}\sum_{x\in {\left\{0,1\right\}}^{n}}{\left(-1\right)}^{f(x)}|x\rangle \left(\frac{|0\rangle -|1\rangle }{\sqrt{2}}\right)\;. \end{array}$$

The appearance of ${\left(-1\right)}^{f(x)}$ in Equation (15) is due to the fact that if $|y\rangle =\frac{|0\rangle -|1\rangle }{\sqrt{2}}$, then

(16) $$\begin{array}{c} |y⊕f(x)\rangle =\left\{\begin{array}{cc} \left[l\right]\frac{|0\rangle -|1\rangle }{\sqrt{2}} & \mathrm{if}\;f(x)=0\\ \frac{|1\rangle -|0\rangle }{\sqrt{2}} & \mathrm{if}\;f(x)=1 \end{array}\right.\Rightarrow |y⊕f(x)\rangle ={\left(-1\right)}^{f(x)}\left(\frac{|0\rangle -|1\rangle }{\sqrt{2}}\right)\;. \end{array}$$

In view of (9) and (15) becomes

(17) $$\begin{array}{c} |\psi_{2}\rangle =\frac{1}{\sqrt{2^{n}}}\sum_{x\in {\left\{0,1\right\}}^{n}}{\left(-1\right)}^{s\cdot x}|x\rangle \left(\frac{|0\rangle -|1\rangle }{\sqrt{2}}\right)\;, \end{array}$$

which is the state returned back to Alice.

Let us now recall the following well-known equation that gives in a succinct form the result of the application of the Hadamard transformation to an arbitrary *n*-qubit basis ket |x〉 (see [26,27]).

(18) $$\begin{array}{c} H^{⊗n}|x\rangle =\frac{1}{\sqrt{2^{n}}}\sum_{z\in {\left\{0,1\right\}}^{n}}^{}{\left(-1\right)}^{z\cdot x}|z\rangle \;. \end{array}$$

Thus, after Alice receives the registers back, she applies the Hadamard transform to the input register for a second time. Via the use of Equation (18), the resulting state can be written as

(19) $$\begin{array}{ccc} |\psi_{3}\rangle \; & = & \frac{1}{\sqrt{2^{n}}}\sum_{x\in {\left\{0,1\right\}}^{n}}{\left(-1\right)}^{s\cdot x}H^{⊗n}|x\rangle \left(\frac{|0\rangle -|1\rangle }{\sqrt{2}}\right)\\ \; & = & \frac{1}{\sqrt{2^{n}}}\sum_{x\in {\left\{0,1\right\}}^{n}}^{}{\left(-1\right)}^{s\cdot x}\left(\frac{1}{\sqrt{2^{n}}}\sum_{z\in {\left\{0,1\right\}}^{n}}^{}{\left(-1\right)}^{z\cdot x}|z\rangle \right)\left(\frac{|0\rangle -|1\rangle }{\sqrt{2}}\right)\\ \; & = & \frac{1}{2^{n}}\sum_{x\in {\left\{0,1\right\}}^{n}}^{}\sum_{z\in {\left\{0,1\right\}}^{n}}^{}{\left(-1\right)}^{s\cdot x⊕z\cdot x}|z\rangle \left(\frac{|0\rangle -|1\rangle }{\sqrt{2}}\right)\\ \; & = & \frac{1}{2^{n}}\sum_{z\in {\left\{0,1\right\}}^{n}}^{}\sum_{x\in {\left\{0,1\right\}}^{n}}^{}{\left(-1\right)}^{(s⊕z)\cdot x}|z\rangle \left(\frac{|0\rangle -|1\rangle }{\sqrt{2}}\right)=|s\rangle \left(\frac{|0\rangle -|1\rangle }{\sqrt{2}}\right)\; \end{array}$$

The last equation is due to the following fact: if s=z, then $\forall \;x\in {\left\{0,1\right\}}^{n}\;\left(s⊕z\right)\cdot x=0$, otherwise for exactly half of the inputs x the exponent will be 0 and for the remaining half the exponent will be 1. This is typically written in a more concise manner as follows:

(20) $$\begin{array}{c} \sum_{x\in {\left\{0,1\right\}}^{n}}^{}{\left(-1\right)}^{(s⊕z)\cdot x}=2^{n}\delta_{s,z}\;. \end{array}$$

The algorithm terminates with the final measurement of the input register by Alice whereby she obtains the secret key s and concludes the whole process.

## 3. QKD Based on Symmetric Entangled B-V

In this section, the two versions of the proposed symmetric entangled QKD protocol based on the Bernstein-Vazirani algorithm are presented and described in great detail. These are the *fully symmetric* version of the protocol, or **fSEBV** for short, and the *semi-symmetric* version of the protocol, or **sSEBV** for short.

### 3.1. The fSEBV Protocol

**Figure 2:**
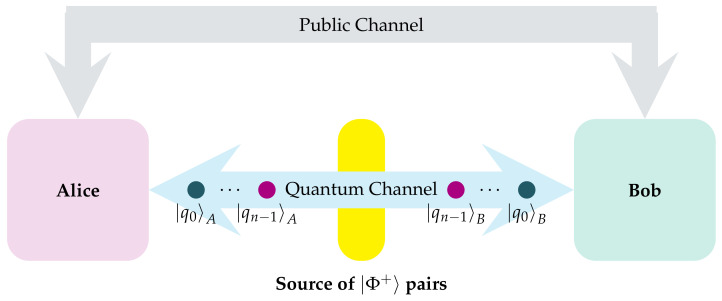
Alice and Bob are spatially separated. A third party, the source, creates *n* pairs of $|\Phi^{+}\rangle$ entangled photons and sends one qubit from every pair to Alice and the other qubit to Bob.

Starting with the fSEBV protocol we consider a slight alteration of the aforementioned Bernstein-Vazirani game. As before, the game starts with the two players Alice and Bob who are spatially separated. This time, instead of using normal qubits in a separable state, they use maximally entangled EPR pairs, and they both share a qubit from each pair. An important rule of the game is that there are no limitations on which entity will actually create the EPR pairs in the first place. The pairs can be created and distributed accordingly by Alice or Bob, or they can be acquired from a third party source. This last situation is depicted in Figure 2. Exactly as in the previous game, the goal of the current game is to acquire a secret key s. However, in this specific protocol symmetry plays a crucial role, as Alice and Bod behave in a perfectly symmetrical way by both having their own secret keys, which they will attempt to input into the system, exactly as in the original algorithm. Alice’s key is denoted by $s_{A}$, Bob’s key by $s_{B}$ and they both take identical actions. Please note that neither Alice nor Bob need apply the Hadamard transform onto their input registers because they are already in the desired even superposition of all basis states, as they are populated by *n* pairs in the $|\Phi^{+}\rangle$ Bell state. In this respect the fSEBV protocol differs from the vanilla Bernstein-Vazirani algorithm.

Following the aforementioned steps of the fSEBV protocol, a valid question may arise regarding what will Alice and Bob acquire after they both apply their starting secret keys $s_{A}$ and $s_{B}$ into their own pieces of the EPR pairs? To provide the answer, let us examine the algorithm more closely. With the help of Equation (7), the initial state of the protocol can be written as

(21) $$\begin{array}{c} |\psi_{0}\rangle ={|\Phi^{+}\rangle }^{⊗n}{|1\rangle }_{A}{|1\rangle }_{B}=\frac{1}{\sqrt{2^{n}}}\sum_{x\in {\left\{0,1\right\}}^{n}}{|x\rangle }_{A}{|x\rangle }_{B}{|1\rangle }_{A}{|1\rangle }_{B}\;. \end{array}$$

Subscripts A and B are consistently used to designate Alice’s and Bob’s registers respectively. Alice and Bob initiate the protocol by applying the Hadamard transform to their output registers, which produces the ensuing state

(22) $$\begin{array}{c} |\psi_{1}\rangle =\frac{1}{\sqrt{2^{n}}}\sum_{x\in {\left\{0,1\right\}}^{n}}{|x\rangle }_{A}{|x\rangle }_{B}{\left(\frac{|0\rangle -|1\rangle }{\sqrt{2}}\right)}_{A}{\left(\frac{|0\rangle -|1\rangle }{\sqrt{2}}\right)}_{B}\;. \end{array}$$

Now, both Alice and Bob can apply their functions on their registers using the standard scheme

(23) $$\begin{array}{c} U_{f}:|x,y\rangle \to |x,y⊕f(x)\rangle \;. \end{array}$$

Consequently, the next state becomes

(24) $$\begin{array}{c} |\psi_{2}\rangle =\frac{1}{\sqrt{2^{n}}}\sum_{x\in {\left\{0,1\right\}}^{n}}{\left(-1\right)}^{f_{A}\left(x\right)}{|x\rangle }_{A}{\left(-1\right)}^{f_{B}\left(x\right)}{|x\rangle }_{B}{\left(\frac{|0\rangle -|1\rangle }{\sqrt{2}}\right)}_{A}{\left(\frac{|0\rangle -|1\rangle }{\sqrt{2}}\right)}_{B}\;. \end{array}$$

At this stage, let us recall that Alice’s and Bob’s functions are

(25) $$\begin{array}{c} f_{A}\left(x\right)=s_{A}\cdot x\;\mathrm{mod}\;2 \end{array}$$

(26) $$\begin{array}{c} f_{B}\left(x\right)=s_{B}\cdot x\;\mathrm{mod}\;2\;, \end{array}$$

where $s_{A}$ and $s_{B}$ are the keys chosen by Alice and Bob, respectively. Based on (24)–(26) can be written as

(27) $$\begin{array}{cc} |\psi_{2}\rangle \; & =\frac{1}{\sqrt{2^{n}}}\sum_{x\in {\left\{0,1\right\}}^{n}}{\left(-1\right)}^{s_{A}\cdot x}{|x\rangle }_{A}{\left(-1\right)}^{s_{B}\cdot x}{|x\rangle }_{B}{\left(\frac{|0\rangle -|1\rangle }{\sqrt{2}}\right)}_{A}{\left(\frac{|0\rangle -|1\rangle }{\sqrt{2}}\right)}_{B}\\ \; & =\frac{1}{\sqrt{2^{n}}}\sum_{x\in {\left\{0,1\right\}}^{n}}{\left(-1\right)}^{s_{A}\cdot x⊕s_{B}\cdot x}{|x\rangle }_{A}{|x\rangle }_{B}{\left(\frac{|0\rangle -|1\rangle }{\sqrt{2}}\right)}_{A}{\left(\frac{|0\rangle -|1\rangle }{\sqrt{2}}\right)}_{B}\\ \; & =\frac{1}{\sqrt{2^{n}}}\sum_{x\in {\left\{0,1\right\}}^{n}}{\left(-1\right)}^{(s_{A}⊕s_{B})\cdot x}{|x\rangle }_{A}{|x\rangle }_{B}{\left(\frac{|0\rangle -|1\rangle }{\sqrt{2}}\right)}_{A}{\left(\frac{|0\rangle -|1\rangle }{\sqrt{2}}\right)}_{B}. \end{array}$$

Subsequently, both Alice and Bob apply the Hadamard transformation to their input registers. This drives the system into the next state, which, by utilizing Equation (18) twice, can be written as

(28) $$\begin{array}{cc} |\psi_{3}\rangle \; & =\frac{1}{\sqrt{2^{n}}}\sum_{x\in {\left\{0,1\right\}}^{n}}{\left(-1\right)}^{(s_{A}⊕s_{B})\cdot x}H^{⊗n}{|x\rangle }_{A}H^{⊗n}{|x\rangle }_{B}{\left(\frac{|0\rangle -|1\rangle }{\sqrt{2}}\right)}_{A}{\left(\frac{|0\rangle -|1\rangle }{\sqrt{2}}\right)}_{B}\\ \; & =\frac{1}{\sqrt{2^{n}}}\sum_{x\in {\left\{0,1\right\}}^{n}}^{}{\left(-1\right)}^{(s_{A}⊕s_{B})\cdot x}\left(\frac{1}{\sqrt{2^{n}}}\sum_{z\in {\left\{0,1\right\}}^{n}}^{}{\left(-1\right)}^{z\cdot x}{|z\rangle }_{A}\right)\left(\frac{1}{\sqrt{2^{n}}}\sum_{w\in {\left\{0,1\right\}}^{n}}^{}{\left(-1\right)}^{w\cdot x}{|w\rangle }_{B}\right)\\ \; & {\left(\frac{|0\rangle -|1\rangle }{\sqrt{2}}\right)}_{A}{\left(\frac{|0\rangle -|1\rangle }{\sqrt{2}}\right)}_{B}\\ \; & =\frac{1}{{\left(\sqrt{2^{n}}\right)}^{3}}\sum_{x\in {\left\{0,1\right\}}^{n}}^{}\sum_{z\in {\left\{0,1\right\}}^{n}}^{}\sum_{w\in {\left\{0,1\right\}}^{n}}^{}{\left(-1\right)}^{(s_{A}⊕s_{B}⊕z⊕w)\cdot x}{|z\rangle }_{A}{|w\rangle }_{B}{\left(\frac{|0\rangle -|1\rangle }{\sqrt{2}}\right)}_{A}{\left(\frac{|0\rangle -|1\rangle }{\sqrt{2}}\right)}_{B}\\ \; & =\frac{1}{{\left(\sqrt{2^{n}}\right)}^{3}}\sum_{z\in {\left\{0,1\right\}}^{n}}^{}\sum_{w\in {\left\{0,1\right\}}^{n}}^{}\sum_{x\in {\left\{0,1\right\}}^{n}}^{}{\left(-1\right)}^{(s_{A}⊕s_{B}⊕z⊕w)\cdot x}{|z\rangle }_{A}{|w\rangle }_{B}{\left(\frac{|0\rangle -|1\rangle }{\sqrt{2}}\right)}_{A}{\left(\frac{|0\rangle -|1\rangle }{\sqrt{2}}\right)}_{B}. \end{array}$$

When $z⊕w=s_{A}⊕s_{B}$, then $\forall x\in {\left\{0,1\right\}}^{n}$, the expression ${\left(-1\right)}^{(s_{A}⊕s_{B}⊕z⊕w)\cdot x}$ becomes ${\left(-1\right)}^{0}=1$ and the sum $\sum_{x\in {\left\{0,1\right\}}^{n}}^{}{\left(-1\right)}^{(s_{A}⊕s_{B}⊕z⊕w)\cdot x}=2^{n}$.

Whenever $z⊕w\neq s_{A}⊕s_{B}$, the sum is just 0 because for exactly half of the inputs x the exponent will be 0 and for the remaining half the exponent will be 1. Hence, one may write that

(29) $$\begin{array}{c} \sum_{x\in {\left\{0,1\right\}}^{n}}^{}{\left(-1\right)}^{(s_{A}⊕s_{B}⊕z⊕w)\cdot x}=2^{n}\delta_{s_{A}⊕s_{B},z⊕w}\;. \end{array}$$

Using Equation (29), and ignoring for the moment the two factors ${\left(\frac{|0\rangle -|1\rangle }{\sqrt{2}}\right)}_{A}$ and ${\left(\frac{|0\rangle -|1\rangle }{\sqrt{2}}\right)}_{B}$, the following two equivalent and symmetric forms can be derived

(30) $$\begin{array}{cc} \; & \sum_{z\in {\left\{0,1\right\}}^{n}}^{}\sum_{w\in {\left\{0,1\right\}}^{n}}^{}\sum_{x\in {\left\{0,1\right\}}^{n}}^{}{\left(-1\right)}^{(s_{A}⊕s_{B}⊕z⊕w)\cdot x}{|z\rangle }_{A}{|w\rangle }_{B}=2^{n}\sum_{z\in {\left\{0,1\right\}}^{n}}^{}{|z\rangle }_{A}{|s_{A}⊕s_{B}⊕z\rangle }_{B}\;, \end{array}$$

and

(31) $$\begin{array}{cc} \; & \sum_{w\in {\left\{0,1\right\}}^{n}}^{}\sum_{z\in {\left\{0,1\right\}}^{n}}^{}\sum_{x\in {\left\{0,1\right\}}^{n}}^{}{\left(-1\right)}^{(s_{A}⊕s_{B}⊕z⊕w)\cdot x}{|z\rangle }_{A}{|w\rangle }_{B}=2^{n}\sum_{w\in {\left\{0,1\right\}}^{n}}^{}{|s_{A}⊕s_{B}⊕w\rangle }_{A}{|w\rangle }_{B}\;. \end{array}$$

By combining (28) with (30) and (31), state $|\psi_{3}\rangle$ can be written in two different ways:

(32) $$\begin{array}{ccc} |\psi_{3}\rangle \; & = & \frac{1}{\sqrt{2^{n}}}\sum_{z\in {\left\{0,1\right\}}^{n}}^{}{|z\rangle }_{A}{|s_{A}⊕s_{B}⊕z\rangle }_{B}{\left(\frac{|0\rangle -|1\rangle }{\sqrt{2}}\right)}_{A}{\left(\frac{|0\rangle -|1\rangle }{\sqrt{2}}\right)}_{B}\\ \; & = & \frac{1}{\sqrt{2^{n}}}\sum_{w\in {\left\{0,1\right\}}^{n}}^{}{|s_{A}⊕s_{B}⊕w\rangle }_{A}{|w\rangle }_{B}{\left(\frac{|0\rangle -|1\rangle }{\sqrt{2}}\right)}_{A}{\left(\frac{|0\rangle -|1\rangle }{\sqrt{2}}\right)}_{B}. \end{array}$$

Finally, Alice and Bob measure their EPR pairs in the input registers, obtaining

(33) $$\begin{array}{c} |\psi_{4}\rangle ={|z_{0}\rangle }_{A}{|s_{A}⊕s_{B}⊕z_{0}\rangle }_{B}={|s_{A}⊕s_{B}⊕w_{0}\rangle }_{A}{|w_{0}\rangle }_{B}\;,\;\mathrm{for}\;\mathrm{some}\;z_{0},w_{0}\in {\left\{0,1\right\}}^{n}\;. \end{array}$$

Please note that in general $z_{0}\neq w_{0}$. The quantum part of the protocol is now complete. The final secret key is the string $s_{A}⊕s_{B}⊕z_{0}$ that Bob measured in his input register. In the highly unlikely event that $|s_{A}⊕s_{B}⊕z_{0}\rangle ={|0\rangle }^{⊗n}$, Bob should inform Alice through the use of the public channel that the whole procedure must be repeated once again, since such a key is clearly unacceptable. However, for a *n*-bit key the probability of this happening is negligible, specifically $\frac{1}{2^{n}}$, which rapidly tends to 0 as n→∞. Hence, it may be safely assumed that Bob possesses a viable secret key, namely $s_{A}⊕s_{B}⊕z_{0}$. Now the final step is for Alice to obtain the secret key too. This is easily achieved by simply having Bob publicly announce his tentative secret key $s_{B}$ to Alice via the use of the public channel. Alice, who has measured the binary string $z_{0}$ and she is already aware of her initial secret key $s_{A}$, can easily obtain the final key, by simply calculating the XOR of $s_{A}$, her measurement $z_{0}$ and Bob’s initial key $s_{B}$, which she learns from the public channel. This concludes the fSEBV protocol.

The symmetry inherent in this protocol, enables the seamless reversal of roles. The protocol, as stated above, grants the initiative to Bob: it is his measurement $s_{A}⊕s_{B}⊕z_{0}$ that produces the secret key and it is his task to send his initial key $s_{B}$ to Alice, in order to successfully complete the procedure. It is equally feasible to have Alice instead of Bob drive the whole process by taking her measurement $s_{A}⊕s_{B}⊕w_{0}$ to be the secret key, as shown in (31). In such an implementation of the fSEBV protocol, Alice must reveal her initial key $s_{A}$ to Bob via the public channel.

During the transmission of Bob’s key $s_{B}$ using a public channel, any potential eavesdropper, namely Eve, does not gain any advantage by listening to the public channel. Due to the fact that she is oblivious of $z_{0}$ and $s_{A}$, she has no way of knowing or computing the final secret key. Hence, the fSEBV protocol ensures that if Alice and Bob can create their keys using a random number generator, in order to avoid possible patterns in the keys, Eve will be left with $2^{n}$ different combinations to test in order to find the secret key.

**Figure 3:**
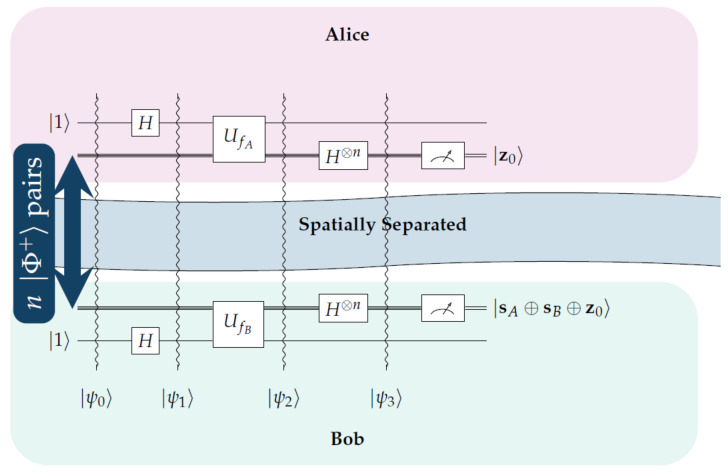
This figures gives a schematic representation of the proposed protocol.

The steps of the protocol from Alice’s and Bob’s side are shown below in an algorithmic manner. Figure 3 depicts the protocol graphically in the form of a quantum circuit.
**Protocol fSEBV:** Alice’s actionsAlice’s input register is populated with entangled qubits  • Alice’s output register is set to |1〉
 • Alice applies the Hadamard transform to her output register  • Alice applies her tentative key $s_{A}$
 • Alice applies the Hadamard transform to her input register  • Alice measures her input register to find the random binary string $z_{0}$
 • Alice receives information from Bob whether the process was a success or must be repeated  • If the procedure was successful, Alice receives from Bob his key $s_{B}$ and, by already knowing $s_{A}$ and $z_{0}$, she computes the final key $s_{A}⊕s_{B}⊕z_{0}$



**Protocol fSEBV:** Bob’s actions• Bob’s input register is populated with entangled qubits • Bob’s output register is set to |1〉
• Bob applies the Hadamard transform to his output register • Bob applies his tentative key $s_{B}$
• Bob applies the Hadamard transform to his input register • Bob measures his input register to find the final secret key $s_{A}⊕s_{B}⊕z_{0}$
• In the unlikely event that $|s_{A}⊕s_{B}⊕z_{0}\rangle ={|0\rangle }^{⊗n}$, Bob informs Alice that the process must be repeated from the start • Otherwise Bob communicates his tentative key $s_{B}$ to Alice via the public channel 


### 3.2. The sSEBV Protocol

The sSEBV protocol explores a special but important case of the fSEBV protocol, which differs from the latter in one important aspect. Alice possesses her random initial key $s_{A}$, but Bob’s key $s_{B}$ is not a random binary string anymore; it is specifically taken to be 0=0⋯0. Essentially, sSEBV protocol answers the question of what will happen, if one of the players, either Alice or Bob, decides not to send a key. As before Alice and Bob are spatially separated and they both share *n* EPR pairs. In this variant, Alice and Bod behave in a semi-symmetrical way. Alice still uses her random initial key $s_{A}$, but Bob is obliged to use 0 as his initial key.

In this case, by using Equation (7), it can seen that the initial state of the system is the following

(34) $$\begin{array}{c} |\psi_{0}\rangle ={|\Phi^{+}\rangle }^{⊗n}{|1\rangle }_{A}{|1\rangle }_{B}=\frac{1}{\sqrt{2^{n}}}\sum_{x\in {\left\{0,1\right\}}^{n}}{|x\rangle }_{A}{|x\rangle }_{B}{|1\rangle }_{A}{|1\rangle }_{B}\;. \end{array}$$

Similarly, Alice and Bob initiate the protocol by applying the Hadamard transform to their output registers, which produces the ensuing state

(35) $$\begin{array}{c} |\psi_{1}\rangle =\frac{1}{\sqrt{2^{n}}}\sum_{x\in {\left\{0,1\right\}}^{n}}{|x\rangle }_{A}{|x\rangle }_{B}{\left(\frac{|0\rangle -|1\rangle }{\sqrt{2}}\right)}_{A}{\left(\frac{|0\rangle -|1\rangle }{\sqrt{2}}\right)}_{B}\;. \end{array}$$

Next Alice and Bob apply their corresponding functions on their registers via the standard scheme

(36) $$\begin{array}{c} U_{f}:|x,y\rangle \to |x,y⊕f(x)\rangle \;, \end{array}$$

only now the situation is quite different because Bob must necessarily use 0:

(37) $$\begin{array}{c} f_{A}\left(x\right)=s_{A}\cdot x\;\mathrm{mod}\;2 \end{array}$$

(38) $$\begin{array}{c} f_{B}\left(x\right)=0\cdot x\;\mathrm{mod}\;2=0\;. \end{array}$$

In view of Equations (37) and (38), the next state becomes

(39) $$\begin{array}{cc} |\psi_{2}\rangle \; & =\frac{1}{\sqrt{2^{n}}}\sum_{x\in {\left\{0,1\right\}}^{n}}{\left(-1\right)}^{f_{A}\left(x\right)}{|x\rangle }_{A}{\left(-1\right)}^{0}{|x\rangle }_{B}{\left(\frac{|0\rangle -|1\rangle }{\sqrt{2}}\right)}_{A}{\left(\frac{|0\rangle -|1\rangle }{\sqrt{2}}\right)}_{B}\\ \; & =\frac{1}{\sqrt{2^{n}}}\sum_{x\in {\left\{0,1\right\}}^{n}}{\left(-1\right)}^{s_{A}\cdot x}{|x\rangle }_{A}{|x\rangle }_{B}{\left(\frac{|0\rangle -|1\rangle }{\sqrt{2}}\right)}_{A}{\left(\frac{|0\rangle -|1\rangle }{\sqrt{2}}\right)}_{B}\;. \end{array}$$

Subsequently, both Alice and Bob apply the Hadamard transformation to their input registers. Taking into account Equation (18), one can see that their combined actions drive the system into the next state

(40) $$\begin{array}{cc} |\psi_{3}\rangle \; & =\frac{1}{\sqrt{2^{n}}}\sum_{x\in {\left\{0,1\right\}}^{n}}{\left(-1\right)}^{s_{A}\cdot x}H^{⊗n}{|x\rangle }_{A}H^{⊗n}{|x\rangle }_{B}{\left(\frac{|0\rangle -|1\rangle }{\sqrt{2}}\right)}_{A}{\left(\frac{|0\rangle -|1\rangle }{\sqrt{2}}\right)}_{B}\\ \; & =\frac{1}{\sqrt{2^{n}}}\sum_{x\in {\left\{0,1\right\}}^{n}}^{}{\left(-1\right)}^{s_{A}\cdot x}\left(\frac{1}{\sqrt{2^{n}}}\sum_{z\in {\left\{0,1\right\}}^{n}}^{}{\left(-1\right)}^{z\cdot x}{|z\rangle }_{A}\right)\left(\frac{1}{\sqrt{2^{n}}}\sum_{w\in {\left\{0,1\right\}}^{n}}^{}{\left(-1\right)}^{w\cdot x}{|w\rangle }_{B}\right)\\ \; & {\left(\frac{|0\rangle -|1\rangle }{\sqrt{2}}\right)}_{A}{\left(\frac{|0\rangle -|1\rangle }{\sqrt{2}}\right)}_{B}\\ \; & =\frac{1}{{\left(\sqrt{2^{n}}\right)}^{3}}\sum_{x\in {\left\{0,1\right\}}^{n}}^{}\sum_{z\in {\left\{0,1\right\}}^{n}}^{}\sum_{w\in {\left\{0,1\right\}}^{n}}^{}{\left(-1\right)}^{(s_{A}⊕z⊕w)\cdot x}{|z\rangle }_{A}{|w\rangle }_{B}{\left(\frac{|0\rangle -|1\rangle }{\sqrt{2}}\right)}_{A}{\left(\frac{|0\rangle -|1\rangle }{\sqrt{2}}\right)}_{B}\\ \; & =\frac{1}{{\left(\sqrt{2^{n}}\right)}^{3}}\sum_{z\in {\left\{0,1\right\}}^{n}}^{}\sum_{w\in {\left\{0,1\right\}}^{n}}^{}\sum_{x\in {\left\{0,1\right\}}^{n}}^{}{\left(-1\right)}^{(s_{A}⊕z⊕w)\cdot x}{|z\rangle }_{A}{|w\rangle }_{B}{\left(\frac{|0\rangle -|1\rangle }{\sqrt{2}}\right)}_{A}{\left(\frac{|0\rangle -|1\rangle }{\sqrt{2}}\right)}_{B}. \end{array}$$

When $z⊕w=s_{A}$, then $\forall x\in {\left\{0,1\right\}}^{n}$, the expression ${\left(-1\right)}^{(s_{A}⊕z⊕w)\cdot x}$ becomes ${\left(-1\right)}^{0}=1$ and the sum $\sum_{x\in {\left\{0,1\right\}}^{n}}^{}{\left(-1\right)}^{(s_{A}⊕z⊕w)\cdot x}=2^{n}$. Whenever $z⊕w\neq s_{A}$, the sum is just 0 because for exactly half of the inputs x the exponent will be 0 and for the remaining half the exponent will be 1. Therefore, again one may write that

(41) $$\begin{array}{c} \sum_{x\in {\left\{0,1\right\}}^{n}}^{}{\left(-1\right)}^{(s_{A}⊕z⊕w)\cdot x}=2^{n}\delta_{s_{A},z⊕w}\;. \end{array}$$

Using Equation (41), and ignoring for the moment the two factors ${\left(\frac{|0\rangle -|1\rangle }{\sqrt{2}}\right)}_{A}$ and ${\left(\frac{|0\rangle -|1\rangle }{\sqrt{2}}\right)}_{B}$, the following two equivalent and symmetric forms can be derived

(42) $$\begin{array}{cc} \; & \sum_{z\in {\left\{0,1\right\}}^{n}}^{}\sum_{w\in {\left\{0,1\right\}}^{n}}^{}\sum_{x\in {\left\{0,1\right\}}^{n}}^{}{\left(-1\right)}^{(s_{A}⊕s_{B}⊕z⊕w)\cdot x}{|z\rangle }_{A}{|w\rangle }_{B}=2^{n}\sum_{z\in {\left\{0,1\right\}}^{n}}^{}{|z\rangle }_{A}{|s_{A}⊕z\rangle }_{B}\;, \end{array}$$

and

(43) $$\begin{array}{cc} \; & \sum_{w\in {\left\{0,1\right\}}^{n}}^{}\sum_{z\in {\left\{0,1\right\}}^{n}}^{}\sum_{x\in {\left\{0,1\right\}}^{n}}^{}{\left(-1\right)}^{(s_{A}⊕z⊕w)\cdot x}{|z\rangle }_{A}{|w\rangle }_{B}=2^{n}\sum_{w\in {\left\{0,1\right\}}^{n}}^{}{|s_{A}⊕w\rangle }_{A}{|w\rangle }_{B}\;. \end{array}$$

By combining (40) with (42) and (43), state $|\psi_{3}\rangle$ can be written in two different ways:

(44) $$\begin{array}{ccc} |\psi_{3}\rangle \; & = & \frac{1}{\sqrt{2^{n}}}\sum_{z\in {\left\{0,1\right\}}^{n}}^{}{|z\rangle }_{A}{|s_{A}⊕z\rangle }_{B}{\left(\frac{|0\rangle -|1\rangle }{\sqrt{2}}\right)}_{A}{\left(\frac{|0\rangle -|1\rangle }{\sqrt{2}}\right)}_{B}\\ \; & = & \frac{1}{\sqrt{2^{n}}}\sum_{w\in {\left\{0,1\right\}}^{n}}^{}{|s_{A}⊕w\rangle }_{A}{|w\rangle }_{B}{\left(\frac{|0\rangle -|1\rangle }{\sqrt{2}}\right)}_{A}{\left(\frac{|0\rangle -|1\rangle }{\sqrt{2}}\right)}_{B}. \end{array}$$

Now, when Alice and Bob measure their input registers, they will obtain

(45) $$\begin{array}{c} |\psi_{4}\rangle ={|z_{0}\rangle }_{A}{|s_{A}⊕z_{0}\rangle }_{B}={|s_{A}⊕w_{0}\rangle }_{A}{|w_{0}\rangle }_{B}\;,\;\mathrm{for}\;\mathrm{some}\;z_{0},w_{0}\in {\left\{0,1\right\}}^{n}\;. \end{array}$$

As in the fSEBV protocol, here also holds that $z_{0}\neq w_{0}$ in general. This time, there are two ways in which the final part of the protocol can unfold. One way, exactly like before, is to take Bob’s measurement as the new secret key. The other, equally viable choice, is to take Alice’s initial key $s_{A}$ as the final secret key. In that case Alice must publicly announce $z_{0}$ to Bob via a public channel, so that he can compute $s_{A}$. This is a suitable choice in cases where, for whatever reason, Alice must set the secret key herself, not wanting to leave anything to chance. In that way she may securely communicate her chosen key to Bob. As before, during the transmission of Alice’s measurement $z_{0}$ using a public channel, Eve does not gain any advantage by eavesdropping on their communication. Due to the fact that she is oblivious to $s_{A}$, she has no way of knowing or computing the final secret key. Hence, the sSEBV protocol also ensures that if Alice devises her key using a random number generator, in order to avoid possible patterns in the keys, Eve will be left with $2^{n}$ different combinations to test in order to find the secret key.

The detailed actions for the implementation of the sSEBV protocol from Alice’s and Bob’s side are given below. Although the sSEBV protocol is not perfectly symmetric, reversal of Alice’s and Bob’s roles is still trivially easy. As can be seen from the following description, not only is Alice the one to choose the secret key, but it is also she that sends the final measurement $z_{0}$ to Bob so that he can successfully derive the secret key. It is equally feasible to have Bob instead of Alice choose the secret key and have Alice use 0 in the first stage. In such a realization of the sSEBV protocol, Bob must also reveal his final measurement $w_{0}$ to Alice via the public channel.

## 4. Examples Illustrating the Operation of the Protocols

**Figure 4:**
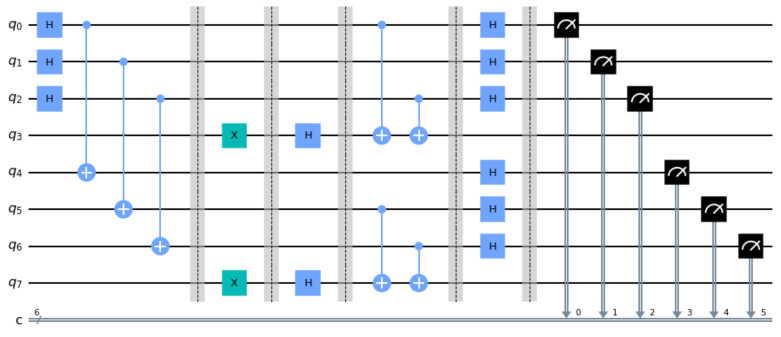
The circuit for the fSEBV protocol.

This section presents and analyzes two small scale but detailed examples in order to illustrate the operation of the fSEBV and sSEBV protocols in practice. The fSEBV and sSEBV protocols were simulated using IBM’s *Qiskit* open source SDK [28]. Specifically, the Aer provider using the high performance *qasm* simulator for simulating quantum circuits [29] in its default settings was used. Please note that during our tests it was not possible to simulate in Qiskit Alice and Bob being spatially separated or a third party source providing the entangled EPR pairs. So these important assumptions cannot be accurately reflected in the simulation and for that reason the examples do not represent a real life environment. As a result Alice and Bob appear in the same circuit. Specifically, Alice’s input register consists of the qubits $|q_{2}q_{1}q_{0}\rangle$ and her output register is $|q_{3}\rangle$. Symmetrically, Bob’s input register consists of the qubits $|q_{6}q_{5}q_{4}\rangle$ and his output register is $|q_{7}\rangle$. Moreover, the entangled EPR pairs are created by the circuit itself. This is depicted in Figure 4, where in the initial stage of the corresponding circuits Hadamard and CNOT gates are used to populate Alice’s and Bob’s input registers with entangled EPR pairs, exactly as explained in Section 2.
**Protocol sSEBV:** Alice’s actions
• Alice’s input register is populated with entangled qubits 
 • Alice’s output register is set to |1〉
 • Alice applies the Hadamard transform to her output register
 • Alice applies her chosen key $s_{A}$
 • Alice applies the Hadamard transform to her input register
 • Alice measures her input register to find the random binary string $z_{0}$
 • Alice announces the binary string $z_{0}$ to Bob via the public channel 


**Protocol sSEBV:**Bob’s actions• Bob’s input register is populated with entangled qubits • Bob’s output register is set to |1〉
• Bob applies the Hadamard transform to his output register • Bob applies his key 0
• Bob applies the Hadamard transform to his input register • Bob measures his input register to find the binary string $s_{A}⊕z_{0}$
• Bob receives $z_{0}$ and computes the key $s_{A}$


### 4.1. Example for the fSEBV Protocol

In this example it is assumed that $s_{A}=101$ and $s_{B}=110$. The resulting circuit in displayed in Figure 4.

**Figure 5:**
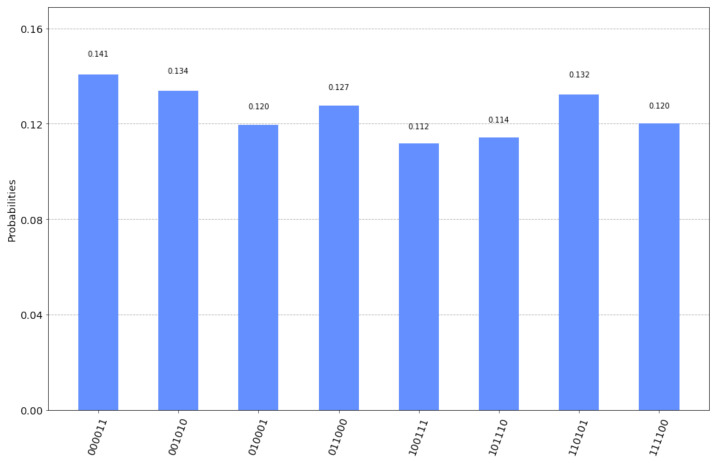
The possible outcomes of the measurement and their corresponding probabilities for the circuit in Figure 4.

The final measurement by Alice and Bob will produce one of the 8 outcomes shown in Figure 5 along with their corresponding probabilities as given by running the qasm simulator for 2048 shots. A simple inspection of the possible outcomes confirms Equation (33). This is because every possible outcome can be written either as ${|z_{0}\rangle }_{A}{|s_{A}⊕s_{B}⊕z_{0}\rangle }_{B}$ or as ${|s_{A}⊕s_{B}⊕w_{0}\rangle }_{A}{|w_{0}\rangle }_{B}$, for some, generally different, $z_{0},w_{0}\in {\left\{0,1\right\}}^{n}$. Hence, Bob, after measuring (and accepting) the secret key $s_{A}⊕s_{B}⊕z_{0}$, just needs to send his secret key $s_{B}=110$ to Alice so that she too can derive the secret key.

To avoid any confusion, we clarify that the measurements shown in Figure 5 depict both Alice’s and Bob’s input registers as $|q_{6}q_{5}q_{4}q_{2}q_{1}q_{0}\rangle$. In particular, every one of the eight possible outcomes is shown along with the probability of measuring this outcome, as computed by the qasm simulator. The three most significant bits represent Bob’s measurement or ${|s_{A}⊕s_{B}⊕z_{0}\rangle }_{B}$ and the three least significant bits represent Alice’s measurement or ${|z_{0}\rangle }_{A}$. Thus, for this specific example, if Bob announces his initial key $s_{B}=110$ to Alice, and Alice performs a XOR operation upon her measurement with Bob’s initial key and her own initial key $s_{A}=101$, then Alice will obtain Bob’s final measurement, which is the secret key.

### 4.2. Example for the sSEBV Protocol

**Figure 6:**
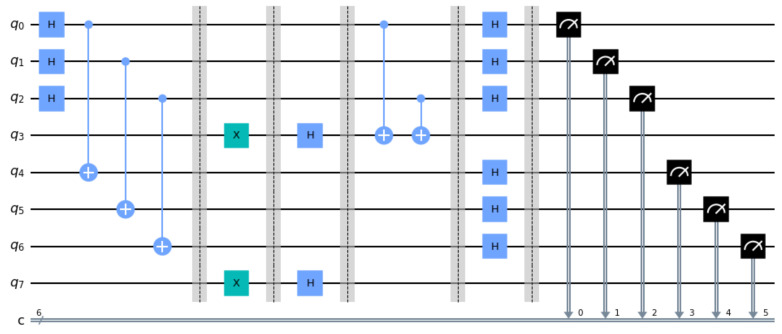
The circuit for the sSEBV protocol.

In this example too, the entangled EPR pairs are created by the circuit itself. In the initial stage of the corresponding circuits Hadamard and CNOT gates are used to populate Alice’s and Bob’s input registers with entangled EPR pairs, as explained in Section 2. Moreover, it is assumed that $s_{A}=101$ and $s_{B}=000$. The resulting circuit in displayed in Figure 6.

**Figure 7:**
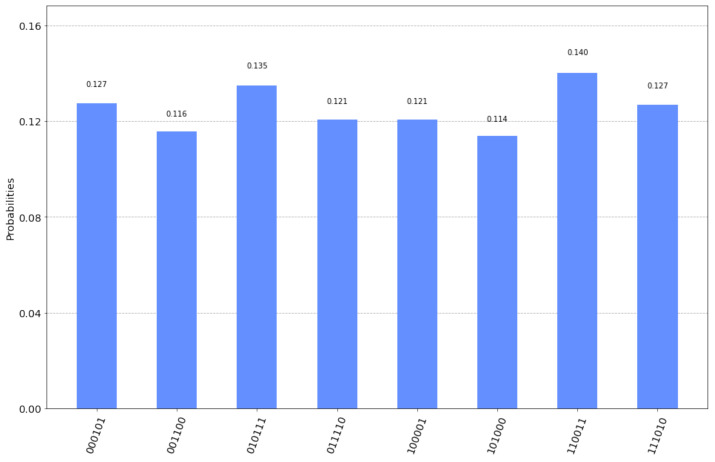
The possible outcomes of the measurement and their corresponding probabilities for the circuit in Figure 6.

This time the final measurement by Alice and Bob will produce one of the 8 outcomes shown in Figure 7 along with their corresponding probabilities as given by running the qasm simulator for 2048 shots. As noted in the previous case, it suffices to inspect the possible outcomes in order to confirm Equation (45). Now the correct interpretation of the outcomes means viewing them either as ${|z_{0}\rangle }_{A}{|s_{A}⊕z_{0}\rangle }_{B}$ or as ${|s_{A}⊕w_{0}\rangle }_{A}{|w_{0}\rangle }_{B}$, for some, generally different, $z_{0},w_{0}\in {\left\{0,1\right\}}^{n}$. Hence, Alice, after making her final measurement and finding a random binary string $z_{0}$, she just needs to send $z_{0}$ to Bob. Then Bob will be able to derive Alice’s chosen secret key $s_{A}=101$.

Again, all of the eight possible outcomes are shown along with the probability of measuring each one of them, as computed by the qasm simulator. The measurements shown in Figure 7 depict both Alice’s and Bob’s input registers as $|q_{6}q_{5}q_{4}q_{2}q_{1}q_{0}\rangle$, that is the three most significant bits represent Bob’s measurement or ${|s_{A}⊕z_{0}\rangle }_{B}$ and the three least significant bits represent Alice’s measurement or ${|z_{0}\rangle }_{A}$. In this specific example, if Alice announces her measurement ${|z_{0}\rangle }_{A}$ to Bob, and Bob performs a XOR operation upon his measurement, with Alice’s measurement, then Bob will obtain the secret key $s_{A}=101$ chosen by Alice.

## 5. Discussion and Conclusions

QKD protocols have surely proved by now that they are the future of key distribution. Their advantage stems from the fact that they allow us to harness the power of quantum-mechanics and nature’s own laws, without having to rely on the complexity of certain mathematical problems. In this paper, we tried to further expand the field of quantum cryptography, by proposing a novel use for the Bernstein-Vazirani algorithm as a symmetrical entanglement-based QKD protocol, coming in two flavors.

These two flavors differ on the degree of symmetry employed by the protocol. In the fully symmetric variant, Alice and Bob take completely identical actions. This variant has the ability to create a totally new and original key, a key that both Alice and Bob were initially oblivious of. This can be useful in many situations as it ensures an additional advantage security wise. Furthermore, it provides a degree of fairness, by putting both parties on an equal footing, in the sense that neither Alice nor Bob can solely determine the secret key.

On the other hand, the semi-symmetric variant, which can technically be viewed as a special case of the first protocol, deviates from this symmetry. In effect, the semi-symmetric protocol answers the question of what will happen if one of the two players wants to specify the secret key. In the presentation given in Section 3 it was Alice that chose the secret key, but it is trivial to adjust the protocol so that Bob can be the party to decide the secret key. This protocol can be useful in situations where a specific key must be chosen by either Alice or Bob, and this key must be securely transmitted to the other party.

Additionally, we demonstrated two small scale but comprehensive examples, illustrating the operation of the two protocols in practice. Finally, we explained the protocols strength against an eavesdropping attack by Eve. Both variants exhibit the inherent robustness of entanglement-based protocols against Eve’s attacks, as originally described by Ekert. Moreover, the use of extra inputs in order to acquire the final secret key, adds another layer of security.

In closing, we remark that we also believe that the rest of the old quantum algorithms, such as the Deutsch-Jozsa algorithm and Simon’s periodicity algorithm, can all be implemented as a symmetrical entanglement-based QKD protocols, posing a viable suggestion for future work, along with the performance of these proposals against different quantum attacks. 

## Figures and Tables

**Table 1:** Alice must communicate with Bob *n* times in order find the secret key s.

The Evolution of the Bernstein-Vazirani Game	
**Alice’s Input x**	**Bob’s Response**
x=10⋯00	$s_{n-1}$
⋮	⋮
x=00⋯10	$s_{1}$
x=00⋯01	$s_{0}$

## Data Availability

Data sharing not applicable.

## References

[1] Shor P.W. (1999). Polynomial-time algorithms for prime factorization and discrete logarithms on a quantum computer. SIAM Rev..

[2] Grover L. A fast quantum mechanical algorithm for database search. Proceedings of the Twenty-Eighth Annual ACM Symposium on the Theory of Computing.

[3] Chen L., Chen L., Jordan S., Liu Y.K., Moody D., Peralta R., Perlner R., Smith-Tone D. (2016). Report on POST-Quantum Cryptography.

[4] Bennett C.H., Brassard G. Quantum Cryptography: Public Key Distribution and Coin Tossing. Proceedings of the IEEE International Conference on Computers, Systems, and Signal Processing.

[5] Ekert A.K. (1991). Quantum cryptography based on Bell’s theorem. Phys. Rev. Lett..

[6] Bennett C.H., Brassard G. (1989). Experimental quantum cryptography: The dawn of a new era for quantum cryptography: The experimental prototype is working. ACM Sigact News.

[7] Elliott C., Colvin A., Pearson D., Pikalo O., Schlafer J., Yeh H. (2005). Current status of the DARPA quantum network. Quantum Information and Computation III.

[8] Elliott C. (2018). The DARPA quantum network. Quantum Communications and Cryptography.

[9] Peev M., Pacher C., Alléaume R., Barreiro C., Bouda J., Boxleitner W., Debuisschert T., Diamanti E., Dianati M., Dynes J. (2009). The SECOQC quantum key distribution network in Vienna. New J. Phys..

[10] Sasaki M., Fujiwara M., Ishizuka H., Klaus W., Wakui K., Takeoka M., Miki S., Yamashita T., Wang Z., Tanaka A. (2011). Field test of quantum key distribution in the Tokyo QKD Network. Opt. Express.

[11] Liao S.K., Cai W.Q., Liu W.Y., Zhang L., Li Y., Ren J.G., Yin J., Shen Q., Cao Y., Li Z.P. (2017). Satellite-to-ground quantum key distribution. Nature.

[12] Deutsch D., Jozsa R. (1992). Rapid solution of problems by quantum computation. Proc. R. Soc. Lond. Ser. A Math. Phys. Sci..

[13] Bernstein E., Vazirani U. (1997). Quantum Complexity Theory. SIAM J. Comput..

[14] Simon D.R. (1997). On the Power of Quantum Computation. SIAM J. Comput..

[15] Nagata K., Nakamura T., Farouk A. (2017). Quantum Cryptography Based on the Deutsch-Jozsa Algorithm. Int. J. Theor. Phys..

[16] Nagata K., Nakamura T. (2017). Quantum Cryptography, Quantum Communication, and Quantum Computer in a Noisy Environment. Int. J. Theor. Phys..

[17] Nagata K., Nakamura T., Geurdes H., Batle J., Abdalla S., Farouk A. (2017). Secure quantum key distribution based on a special Deutsch-Jozsa algorithm. Asian J. Math. Phys..

[18] Diffie W., Hellman M. (1976). New directions in cryptography. IEEE Trans. Inf. Theory.

[19] Bennett C.H., Brassard G. (2014). Quantum cryptography: Public key distribution and coin tossing. Theor. Comput. Sci..

[20] Aharon N., Silman J. (2010). Quantum dice rolling: A multi-outcome generalization of quantum coin flipping. New J. Phys..

[21] Meyer D.A. (1999). Quantum strategies. Phys. Rev. Lett..

[22] Eisert J., Wilkens M., Lewenstein M. (1999). Quantum games and quantum strategies. Phys. Rev. Lett..

[23] Andronikos T., Sirokofskich A., Kastampolidou K., Varvouzou M., Giannakis K., Singh A. (2018). Finite Automata Capturing Winning Sequences for All Possible Variants of the PQ Penny Flip Game. Mathematics.

[24] Andronikos T., Sirokofskich A. (2021). The Connection between the PQ Penny Flip Game and the Dihedral Groups. Mathematics.

[25] Giannakis K., Theocharopoulou G., Papalitsas C., Fanarioti S., Andronikos T. (2019). Quantum Conditional Strategies and Automata for Prisoners’ Dilemmata under the EWL Scheme. Appl. Sci..

[26] Nielsen M.A., Chuang I.L. (2010). Quantum Computation and Quantum Information.

[27] Mermin N. (2007). Quantum Computer Science: An Introduction.

[28] Qiskit Qiskit Open-Source Quantum Development. https://qiskit.org.

[29] Qasm The Qasm Simulator. https://qiskit.org/documentation/stubs/qiskit.providers.aer.QasmSimulator.html.

